# Disease-specific growth charts of Marfan syndrome in Korea

**DOI:** 10.1186/1687-9856-2015-S1-P30

**Published:** 2015-04-28

**Authors:** Su Jin Kim, Sung Won Park, Younghee Kwun, Dong-Kyu Jin

**Affiliations:** 1 Department of Pediatrcis, Myongji Hospital, Goyang, Korea; 2 Department of Pediatrics, Jeil Hospital, Kwandong University School of Medicine, Seoul, Korea; 3 Department of Pediatrics, Samsung Medical Center, Sungkyunkwan University School of Medicine, Seoul, Korea

## Aims

Understanding the growth pattern in Marfan syndrome (MFS) is useful for predicting adult height and for planning the timing of growth reduction therapy. We have reviewed the growth parameters of patients with MFS in Korea and generated the Korean MFS-specific growth curve for understanding the growth pattern in MFS.

## Methods

Anthropometric data were available from 187 males and 152 females with MFS through a retrospective review of medical records. The standardized growth curves were constructed for weight and height according to gender. Comparisons between MFS patients and the general population were performed using a one-sample T-test.

## Results

Korean MFS patients had similar height and weight compared with the general population at birth. However, linear growth curve of Korean MFS after two years of age showed that the 50th percentile of MFS is above the 97th percentile of normal in both genders. Regarding body mass, although the mean body weight of MFS patients was larger than that of the general population in males and females, the gap of the mean weight curve was small. In the Korean MFS growth curve, the growth pattern and final adult height were nearly analogous to those of the United States (US).

## Conclusions

Korean MFS-specific growth charts showed that an excessive growth pattern began in the early infant period, which was prominent in terms of linear growth compared to body mass. There were no ethnic differences in the growth pattern compared with Western MFS patients.

